# Effects of valproic acid on SOCS-1, SOCS-2, SOCS-3, SOCS-5, SOCS6, and SOCS-7 gene expression and cell growth inhibition in colon carcinoma 

**Published:** 2022

**Authors:** Masumeh Sanaei, Fraidoon Kavoosi, Masoud Safarzadeh

**Affiliations:** 1 *Research Center For Non-Communicable Diseases, Jahrom University of Medical Sciences, Jahrom, Iran*; 2 *Student of Research Committee, Jahrom University of Medical Sciences, Jahrom, Iran*

**Keywords:** Valproic acid, SOCS, Apoptosis, Colon carcinoma

## Abstract

**Aim:**

The current study aimed to investigate the effect of valproic acid (VPA) on SOCS-1, SOCS-2, SOCS-3, SOCS-5, SOCS6, and SOCS-7 gene expression and cell growth inhibition in colon carcinoma IS1, IS2, and IS3 cell lines.

**Background:**

Cancer is a process induced by the accumulation of epigenetic alterations such as DNA methylation and histone deacetylation. The DNA methylation and histone deacetylation of tumor suppressor genes (TSGs) have been shown in various cancers. The methylation and deacetylation of suppressors of the cytokine signaling (SOCS) family, as TSGs, have been demonstrated in numerous cancers. Histone deacetylase inhibitors (HDACIs) have emerged as accessory therapeutic agents for human cancers.

**Methods:**

IS1, IS2, and IS3 cells were cultured and treated with VPA. To determine cell viability, cell apoptosis, and the relative gene expression level, MTT assay, flow cytometry assay, and qRT-PCR, respectively, were performed. A database was established with the SPSS 16.0 software package (SPSS Inc., Chicago, Illinois, USA) for analysis. Data was acquired from three tests and is shown as means ± standard deviations. A significant difference was considered as *p* < 0.05.

**Results:**

VPA changed the expression levels of the SOCS-1, SOCS-2, SOCS-3, SOCS-5, SOCS6, and SOCS-7 genes, by which cell apoptosis was induced and cell growth inhibited in all three cell lines (*p *< 0.0001).

**Conclusion:**

VPA can induce apoptosis through reactivation of SOCS-1, SOCS-2, SOCS-3, SOCS-5, SOCS6, and SOCS-7 gene expression.

## Introduction

 Cancer is a process induced by the accumulation of epigenetic alterations such as DNA methylation and histone deacetylation. The DNA methylation and histone deacetylation of tumor suppressor genes (TSGs) have been shown in various cancers. The TSGs inhibit cancer induction and progression by encoding a group of cell cycle regulatory proteins that inhibit cell cycle progression and/or promote cell apoptosis. These genes may lose their functions and become inactivated because of three principal mechanisms: (i) Homozygous deletion of both alleles leading to loss of function; (ii) Loss of one allele; and (iii) Epigenetic alteration such as the methylation of CpG islands or histone deacetylation resulting in gene silencing (1). Recent studies have shown that the silencing of TSGs, resulting from epigenetic alterations such as deacetylation, is an early event in many human cancers (2). The methylation and deacetylation of suppressors of the cytokine signaling (SOCS) family, as TSGs, have been demonstrated in numerous cancers. This family has eight members, comprising SOCS-1 to SOCS-7 and cytokine-inducible SH2 protein (3). The histone deacetylation of SOCS-1 and SOCS-3 has been indicated in colon cancer, and that of SOCS1 has been indicated in human cervical carcinoma cell lines HeLa, CaSki, and SiHa (4). Histone deacetylase inhibitors (HDACIs) have emerged as accessory therapeutic agents for human cancers, as they can inhibit the activity of specific HDACs, restore the expression of some TSGs, and induce cell growth inhibition and apoptosis. They can be divided into four groups based on their structures: cyclic peptides, hydroximates, aliphatic acids, and benzamides. The apoptotic effect of HDACIs has been shown in various cancers such as non-small cell lung cancer (MV522, A549), leukemia (K562), ovarian cancer (SKOV-3), Jurkat, HT29 colon cancer, and myelogenous leukemia (5). In vitro studies have demonstrated that HDACIs play their apoptotic roles through various mechanisms. Previously, we reported that the histone deacetylase inhibitor valproic acid can reactivate silenced SOCS-1 and SOCS-3, resulting in apoptosis induction in the colon carcinoma SW48 cell line (6). The current study aimed to investigate the effect of valproic acid (VPA) on SOCS-1, SOCS-2, SOCS-3, SOCS-5, SOCS6, and SOCS-7 gene expression and cell growth inhibition in colon carcinoma IS1, IS2, and IS3 cell lines to determine whether valproic acid can reactivate the expression of the mentioned genes in the named cell lines. 

## Methods

**Materials**

Human colon carcinoma IS1, IS2, and IS3 cell lines were purchased from the National Cell Bank of Iran-Pasteur Institute. The VPA and Dulbecco’s modified Eagle’s medium (DMEM) were obtained from Sigma (St. Louis, MO, USA). VPA was dissolved in sterile water to make a work-stock solution. Further concentrations of VPA were obtained by diluting the stock solution. Other necessary materials and kits were purchased as in previous works (7, 8). The IS1, IS2, and IS3 cells were maintained in DMEM supplemented with fetal bovine serum 10% and antibiotics in a humidified atmosphere of 5% CO2 in air at 37 ℃. This work was approved by the Ethics Committee of Jahrom University of Medical Science under code number of IR.JUMS.REC. 1399.118. 

**Cell culture and cell viability**

The IS1, IS2, and IS3 cells were cultured in DMEM supplemented with 10% FBS and antibiotics at 37 °C in 5% CO2 overnight and then seeded into 96-well plates (3× 10^5 ^cells per well). After 24 h, the culture medium was replaced with a medium containing various doses of VPA (0, 1, 5, 10, 25, and 50 μM). The control groups were exposed to an equivalent volume of solvent. After 24 h of treatment, the treated and untreated cells were investigated by MTT assay according to standard protocols to determine cell viability. The MTT assay was achieved as previously described (9, 10).

**Cell apoptosis assay**

To determine IS1, IS2, and IS3 cell apoptosis, cells were cultured at a density of 3 × 10^5^ cells/well and treated with VPA based on IC50 values indicated in Table 1 for 24 h. The control groups were exposed to an equivalent volume of solvent. Then, the IS1, IS2, and IS3 cells were harvested by trypsinization, washed with cold PBS, and resuspended in binding buffer (1x). Finally, 5 μL of annexin V-FITC solution and 10 μL of PI solution were used according to the protocol. Cells were incubated for 15 minutes at room temperature in the dark and measured with Becton Dickinson FACScan flow cytometry (Becton Dickinson, Heidelberg, Germany). Each experiment was performed in triplicate (11, 12). 

**Real-time Quantitative Reverse Transcription Polymerase Chain Reaction (qRT-PCR)**

To determine the relative expression levels of the SOCS-1, SOCS-2, SOCS-3, SOCS-5, SOCS6, and SOCS-7 genes, qRT-PCR was performed. The IS1, IS2, and IS3 cells (at a density of 3 × 10^5^ cells/well) were treated with VPA, based on IC 50 value, for 24 h. The control groups were exposed to an equivalent volume of solvent. Then qRT-PCR was done as in our previous works (13, 14). The primer sequences are shown in Table 2 (15-19). 

## Results

**Cell viability (MTT assay)**

**Figure1:**
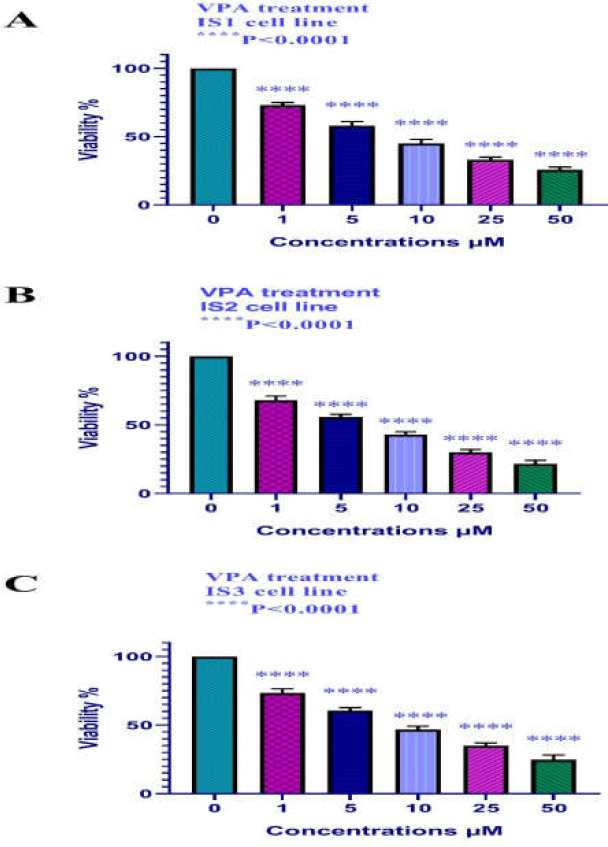
In vitro effects of VPA (0, 1, 5, 10, 25, and 50 μM) on colon carcinoma IS1, IS2, and IS3 cell viability determined by MTT Assay at 24 h. As indicated in figure 1, VPA inhibited the growth of all three cell lines significantly in a dose-dependent manner

The cell viability of the IS1, IS2, and IS3 cells treated with various doses of VPA (0, 1, 5, 10, 25, and 50 μM) was investigated by MTT assay. As shown in Figure 1, VPA induced significant cell growth inhibition (*p *< 0.001). The IC50 value was calculated by Graph pad prism 8 as indicated in Table 1. 

**Figure 2:**
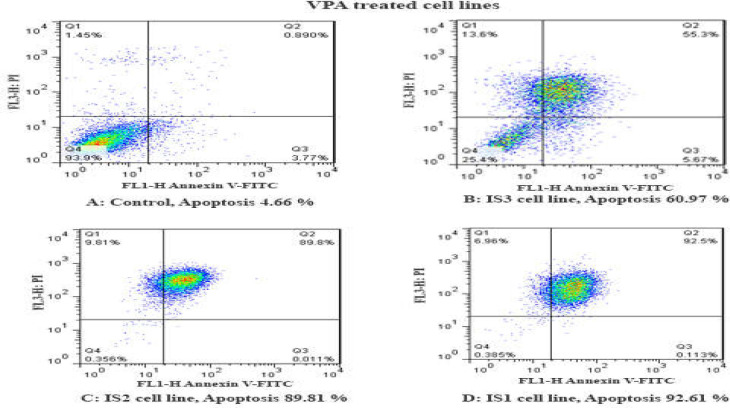
The apoptotic effect of VPA on colon carcinoma IS1, IS2, and IS3 cells versus control groups at 24 h, the control groups were exposed to an equivalent volume of solvent. The VPA induced significant apoptosis. The results were obtained from three independent experiments. Maximal apoptosis was seen in the IS1 cell line after 24 h. Quadrant (Q) 2 and 3, indicates late and primary apoptosis respectively. This two-quadrant was calculated in this graph

**Figure 3:**
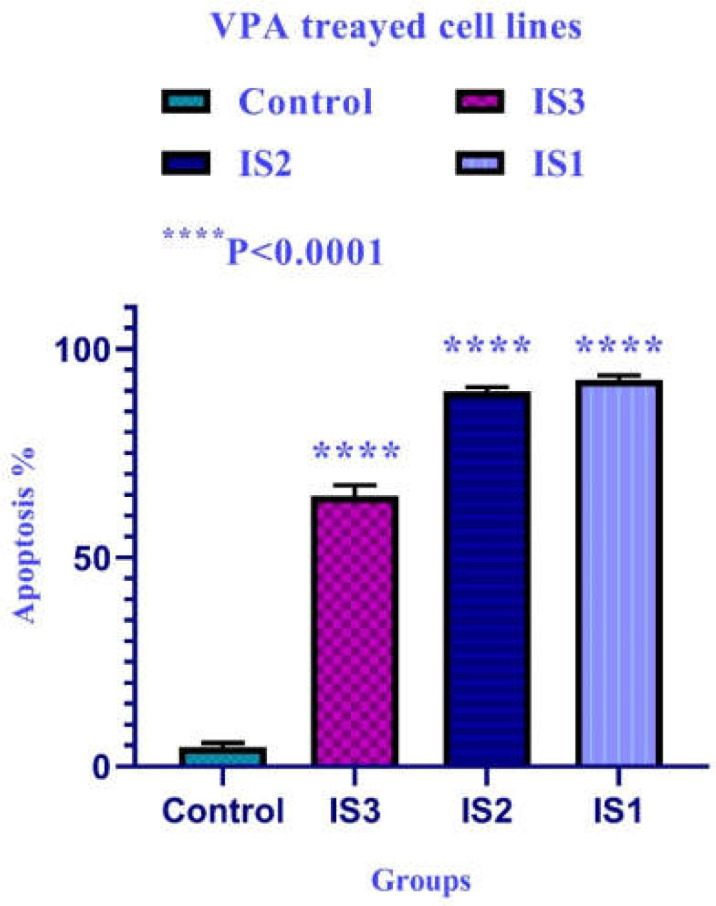
The comparative apoptotic effects of VPA on colon carcinoma IS1, IS2, and IS3 cells). the control groups were exposed to an equivalent volume of solvent. Asterisks (*) indicate significant differences between the treated and untreated control groups. As demonstrated above, Maximal apoptosis was seen in the IS1 cell line after 24 h.

**Table 1:** IC50 values of VPA

Cell line	Duration/Hour	IC50/ μM	LogIC50	R squared
IS1	24	4.509	0.6541	0.8989
IS2	24	5.011	0.6999	0.8690
IS3	24	5.765	0.7608	0.8979

**Table 2:** The Primer Sequences of SOCS-1, SOCS-2, SOCS-3, SOCS-5, SOCS6, SOCS-7, and GAPDH

Primer	Primer sequences (5' to 3')	Product length	Reference
SOCS-1 Forward Reverse	TTTTTCGCCCTTAGCGTGA AGCAGCTCGAAGAGGCAGTC	119 bp	
SOCS-2 Forward Reverse	AGTGTGGTTCATCTGATCG ACATTTGTTAATGGTGAGCCT	162 bp	
SOCS-3 Forward Reverse	GGCCACTCTTCAGCATCTC ATCGTACTGGTCCAGGAACTC	109 bp	
SOCS-5 Forward Reverse	CCTACAGGTGTTCAGTAAGAC CCACACTGTTGAAATACTCATCC	171 bp	
SOCS6 Forward Reverse	ATCACGGAGCTATTGTCTGGA CTGACTCTCATCCTCGGGGA	97 bp	
SOCS-7 Forward Reverse	CTTCAAAATCCGCCTCAGTC CTGAAGGAGACCGGAGAAAA	213 bp	
GAPDH Forward Reverse	TCCCTGAGCTGAACGGGAAG GGAGGAGTGGGTGTCGCTGT	218 bp	

**Cell apoptosis**

To determine cell apoptosis, cells were treated with VPA and then stained using annexin-V-(FITC) and PI as mentioned in the materials and methods. 

 As indicated in Figures 2 and 3, both compounds induced cell apoptosis significantly (*p *< 0.001). 

**Gene expression**

**Figure 4:**
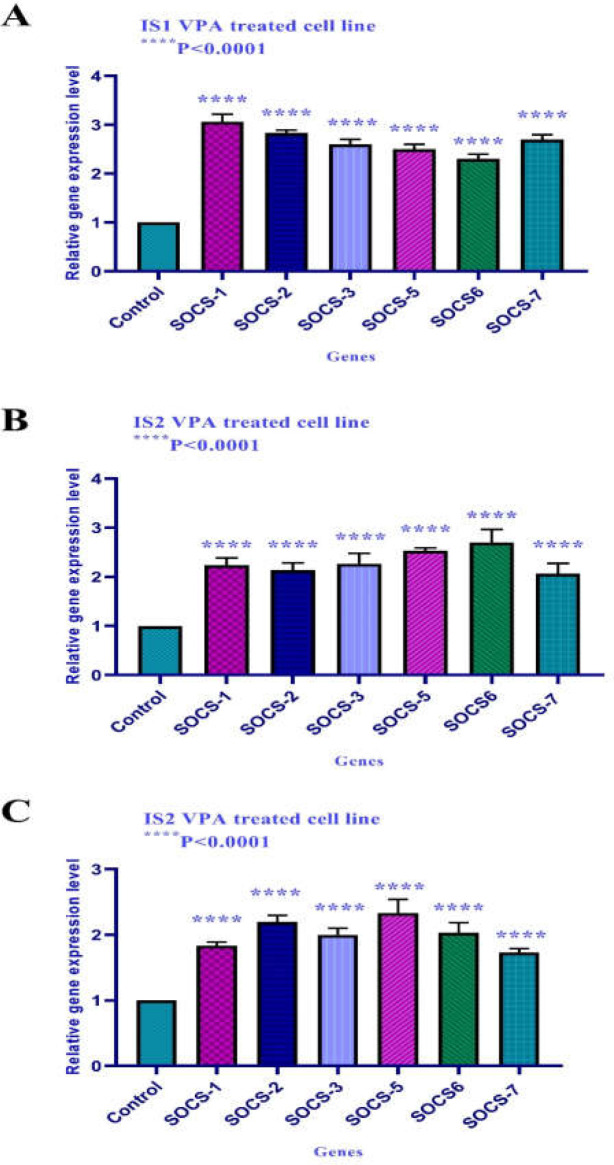
The relative expression level of SOCS-1, SOCS-2, SOCS-3, SOCS-5, SOCS6, and SOCS-7 in the IS1, IS2, and IS3 cell line treated with VPA versus untreated control groups at 24 h, the control groups were exposed to an equivalent volume of solvent. As indicated in this Figure, this compound up-regulated SOCS-1, SOCS-2, SOCS-3, SOCS-5, SOCS6, and SOCS-7 gene expression significantly after 24 h of treatment in IS1, IS2, and IS3 cell lines

The effect of VPA on SOCS-1, SOCS-2, SOCS-3, SOCS-5, SOCS6, and SOCS-7 gene expression was evaluated by quantitative real-time RT-PCR analysis. The results demonstrated that this compound significantly upregulated SOCS-1, SOCS-2, SOCS-3, SOCS-5, SOCS6, and SOCS-7 gene expression after 24 h of treatment in all three cell lines, as indicated in Figure 4.

## Discussion

There are several universal aberrations common to all cancers, one of which is the epigenetic silencing of TSGs, which is thought to be an early driving event in the tumorigenesis and oncogenic process (20). Recent studies have indicated that histone deacetylation of TSGs is involved in tumorigenesis (21). HDACIs represent a new class of anticancer drugs able to induce cell apoptosis and/or cell cycle arrest and influence gene expression by enhancing the acetylation of histones (22). In the current study, it was found that VPA can upregulate SOCS-1, SOCS-2, SOCS-3, SOCS-5, SOCS6, and SOCS-7 genes, resulting in cell growth inhibition and apoptosis induction in the IS1, IS2, and IS3 cell lines. Previously, we reported that valproic acid upregulated SOCS-1 and SOCS-3 gene expression in colon carcinoma SW48 cell line (6). Similarly, it has been reported that the histone deacetylase inhibitor vorinostat induces apoptosis by reactivation of SOCS1 and SOCS3 in polycythemia vera (PV) (23). Furthermore, sodium butyrate treatment increases the transcript levels of SOCS1 and SOCS3 and inhibits cell proliferation in myeloproliferative neoplasms (MPNs) and leukemia (24). Trichostatin A (TSA) is another inhibitor of HDACs. 

Treatment with this compound increases the levels of SOCS1 and SOCS3 in myeloproliferative neoplasms (MPNs) and leukemia (25). Moreover, trichostatin A (TSA) leads to the hyperacetylation of histones associated with SOCS1 and SOCS3 promoters, which significantly downregulates JAK2/STAT3 signaling in colorectal cancer cells. Therefore, TSA may induce SOCS1 and SOCS3 expression by inducing histone modifications and, consequently, inhibits JAK2/STAT3 

signaling in this cancer. These results establish a link between the inhibition of JAK2/STAT3 signaling and the anticancer effect of TSA in the colorectal cancer cell line (26). In myeloproliferative neoplasms (MPNs) and leukemia, sodium butyrate inhibits JAK2/STAT signaling through HDAC8-mediated upregulation of SOCS1 and SOCS3 (24). Finally, current evidence suggests that HDACIs upregulate the SOCSs through JAK2/STAT signaling inhibition. In the current study, JAK2/STAT was not assessed because of budget limitations. Therefore, this evaluation is recommended. 

The results of the current study demonstrate that VPA plays its role through the upregulation of SOCS-1, SOCS-2, SOCS-3, SOCS-5, SOCS6, and SOCS-7 genes, resulting in cell growth inhibition and apoptosis induction in colon carcinoma IS1, IS2, and IS3 cell lines.

## Conflict of interests

The authors declare that they have no conflict of interest.
